# Solitary lower lumbar osteochondroma (spinous process of L3 involvement): a case report

**DOI:** 10.1186/1757-1626-2-9359

**Published:** 2009-12-20

**Authors:** Ebrahim Ghayem Hassankhani

**Affiliations:** 1 Department of Orthopedic and Spine Surgery, Imam Reza University Hospital, Ebnesina Street, Mashhad, 9187895356, Iran

## Abstract

Solitary osteochondromas, which are the most common benign bone tumors of long bones, are rarely found in the vertebral column. A 16-year-old female patient presented with a hard palpable mass at lower lumbar region like a congenital deformity. Plain radiography illustrated a well-defined solid mass arising from the posterior elements of the L3 and ruled out any congenital anomalies. A computed tomography scan further determined a mass that arose from the spinous process of L3. The tumor was excised en bloc through a posterior approach and histopathological examination verified the diagnosis of osteocondroma.

Osteochondromas are rarely found in the spine, when present in the spine, however, have a predilection for cervical or upper thoracic region arising usually from lamina of vertebrae and are rare in lumbosacral region and very rare at spinous process of the vertebrae.

We present a case of osteochondroma locates in lumbar region and spinous process of vertebrae with unusual presentation and was considered clinically as congenital lumbar kyphosis.

## Introduction

Osteochondroma is the most common primary benign bone tumor. However, this tumor rarely involves the spine and even more rarely involves the lower lumbar region [1]. They represent 2% of all tumors and 2.6% of the benign tumors of the spine [2].

Presentation of these tumors in spine is usually circumscribed to the cervical and upper thoracic regions with few tumors presenting in the lumbosacral vertebrae [3-5].

In this report, the osteochondroma was located at the spinous process of L3.

## Case presentation

**Figure 1:**
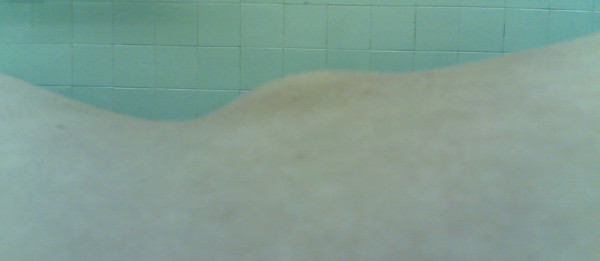
**A 16 years old female with lower lumbar osteochondroma**.

A 16-year-old girl from Khorasan state of IRAN was referred to our spine clinic as a congenital lumbar kyphosis with a palpable non tender mass in the midline of lower lumbar region which was noticed by her parents two years prior to presentation to our hospital (Figure 1).

The examination revealed a hard palpable mass at the lower lumbar region, like a gibbous deformity, without pain or tenderness and with a size approximately 5 × 4 cm. The lesion was not mobile. The overlying skin and the movements of the lumbar were normal. There was no neurological deficit.

His past medical history and family history for similar lesion was negative.

**Figure 2:**
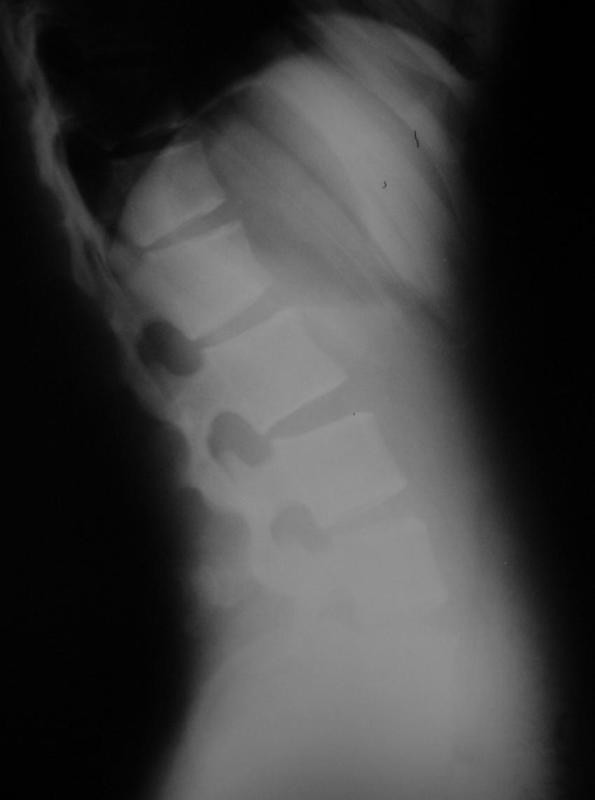
**Lateral plain radiography of lumbar with L3 osteochondroma**.

The initial plain radiographs were no diagnostic. Lumbosacral anteroposterior and lateral plain radiographs was taken again, illustrated a well - defined solid mass arising from the posterior elements of the L3 and rolled out any Congenital anomalies (Figure 2).

**Figure 3:**
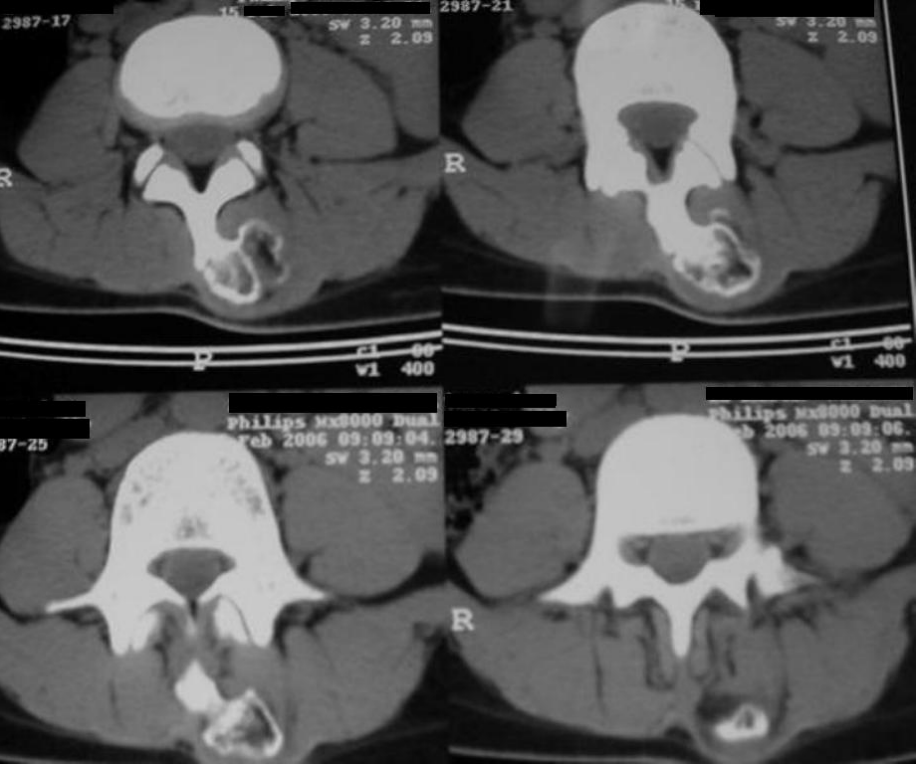
**Axial view of C.T.S of L3 shows osteochondroma of spinous process**.

**Figure 4:**
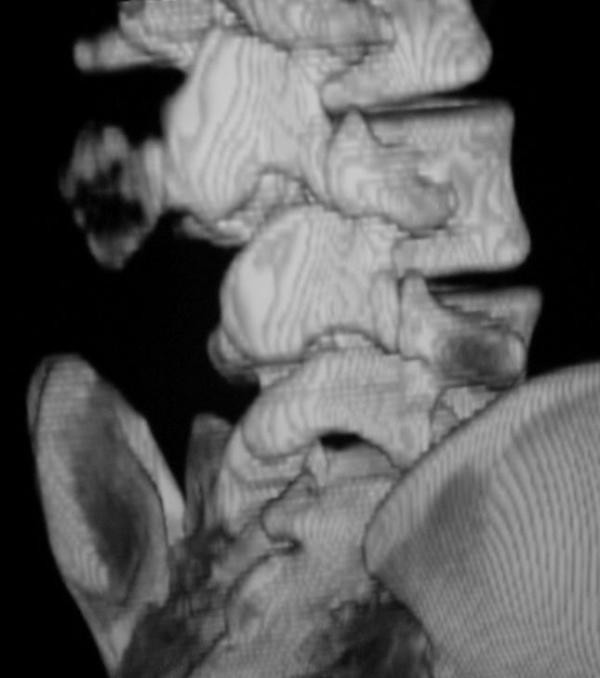
**T.D.CTS of lower lumbar with osteochondroma of spinous process of L3**.

A computed tomography scan (CT) further determined a mass that arose from the spinous process of L3 (Figure 3, and Figure 4).

**Figure 5:**
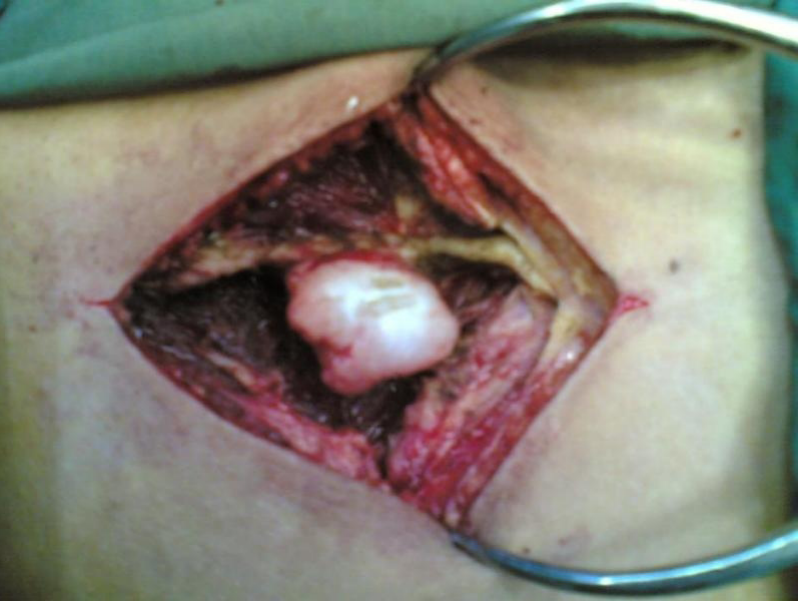
**Intraoperative view of osteochondroma of L3 spinous Process**.

The tumor was excised en bloc through a posterior approach (Figure 5). Histopathological examination verified the diagnosis of osteochondroma. During 19 months follow-up, no recurrence was detected.

## Discussion

Osteochonroma is more frequent in male and patients are usually under the age of 20 [3-5].

From 1-4% of osteochondromas involve the spine and are commonly included in the posterior elements of the vertebrae. Osteochondroma has a predilection for the cervical and upper thoracic regions and, as it is in our case, rarely involve Lumbosacral region of the spine [6].

As this case, a simple painless mass may be the only presenting symptom [7], but if located near neurologic or vascular structures, Cause cord, root and vascular compression that potentially is catastrophic [1,2,6,8]. Some cases of osteochondroma of the spine have been reported with radicular pain, Limitation of the motion and scoliosis [[1,2,6], and [9]].

In all of the reported cases, the localization of osteocondroms are the posterior arch of the vertebrae more commonly the lamina and involvement of the spinous process specially in lumbar region as this case is very rare and has not been reported [7].

Plain radiographs may be nondiagnostic in certain cases, and computed tomography scan is necessary for evaluating the origin, size and characteristic of the tumor [3,5]. In cases with neurologic symptoms, Magnetic resonance imaging is helpful for the demonstration of spinal cord or nerve root compression [10].

Osteochondromas that consist the largest portion of benign bone tumors, usually occur in the long bones and are rarely found in the spine when present in the spine, however, have a predilection for cervical or upper thoracic region arising usually from lamina of vertebrae. Therefore we presented an osteochondroma locate in Lumbar region and spinous process of vertebrae with unusual presentation was considered clinically as Lumbar kyphosis.

## Consent

Written informed consent was obtained from the patient for publication of this case report and accompanying images. A copy of the written consent is available for review by the Editor-in-Chief of this journal.

## Competing interests

The author declares that he has no competing interests.

## References

[B1] Samartzis D Marco RA Osteochondroma of the sacrum: A case report and review of the Literature Spine 2006 31 13 e420 429 10.1097/01.brs.0000220222.63828.d3 16741444

[B2] Carrera JE Castillo PA Molina OM Lumbar Osteochondroma and radicular compression. A case report Acta Orthop Men 2007 21 5 261 266 18159915

[B3] Nejmi K Ali D Nebi Y A Giant cervical osteochondroma Eur J Gen Med 2005 2 3 120 122

[B4] Arasil E Erdem A Yuceer N Osteochondroma of the uppe cervical spine: A case report Spine 1996 21 216 218 10.1097/00007632-199602150-00021 8658258

[B5] Ratliff J Voorhies R Osteochondroma of the C5 Lamina with cord compression: case report and review of the Literature Spine 2000 25 1293 1295 10.1097/00007632-200005150-00017 10806510

[B6] Fiumara E Scarabino T Guglielmi G Bisceglia M D'Angelo V Osteochondroma of the L-5 vertebra: a rare cause of sciatic pain. Case report J Neurosurg 1999 91 Suppl 2 219 222 10505509 10.3171/spi.1999.91.2.0219

[B7] Silber JS Mathur S Ecker M A solitary osteochondroma of the pediatric spine: a case report and review of the literature Am J Orthop 2000 29 9 711 714 11008869

[B8] Woertler K Lindner N Gisheger G Osteochondroma: MR imaging of Tumor - related complications Eur Radiol 2000 10 832 840 10.1007/s003300051014 10823643

[B9] Jose Alcaraz MM Izquierdo NE Santonja GC Osteochondroma of the thoracic spine and scoliosis Spine 2001 26 9 1082 1085 10.1097/00007632-200105010-00019 11337629

[B10] Cirak B kerabulut N Palaoglu S Cervical osteochondlroma as a cause of spinal cord compression in a patient with hereditary multiple exostoses; computed tomography and Magnetic resonance imaging findings Australas Radiol 2002 46 309 311 10.1046/j.1440-1673.2002.01067.x 12196244

